# Comment on “Assessing Effectiveness and Costs in Robot-Mediated Lower Limbs Rehabilitation: A Meta-Analysis and State of the Art”

**DOI:** 10.1155/2018/7634965

**Published:** 2018-11-28

**Authors:** Alberto Esquenazi

**Affiliations:** Professor and Chair Department of Physical Medicine and Rehabilitation, MossRehab/Einsteinm, 60 Township Line Rd., Elkins Park, PA 19027, USA

It is with great interest that we read the article “Assessing Effectiveness and Costs in Robot-Mediated Lower Limbs Rehabilitation: A Meta-Analysis and State of the Art” by Carpino et al. [1], which was published in the *Journal of Healthcare Engineering*, Volume 2018 (ID 7492024). We believe the authors are investigating and discussing a very important topic, i.e., the financial efficiency of modern technology in rehabilitation.

We, however, would like to express our concern about some of their calculations and conclusions.

## 1. Purchasing Costs

Our biggest concern is the indicated purchasing costs. In their calculations, Carpino et al. [1] differentiate between operational machines (commonly referred to as end-effector devices) and wearable robots (commonly referred to as exoskeleton devices) with the goal to compare the financial efficiency of the two classes with each other and with conventional training. The authors have based all their calculations on a purchasing price of €330,000 for a Lokomat (as an example of an exoskeleton) and € 30,000 for a Gait Trainer GT1 (as an example of an end-effector). However, the LokomatPro is a state-of-the-art representative of the exoskeleton group (and at the mentioned price in Italy, it likely includes the optional FreeD feature as well as more than one year of maintenance). The GT1, which they use as the representative of the end-effector device group, has long been replaced by the GTII and further developed into the G-EO, which has a list price of €250,000. Therefore, when comparing a state-of-the-art exoskeleton device to a state-of-the-art end-effector device, the difference in costs is not, as indicated, tenfold, but much smaller. Similarly, the maintenance costs, which in the article are indicated as 10% of the device or €22,500 per year, are actually just below €10,000 per year for the Lokomat based on the data provided by Hocoma device manufacturer and just slightly higher than that quoted by the G-EO manufacturer. We cordially invite the authors to repeat their calculations with these numbers.

## 2. Human Resources: Establishing Efficient Settings

Secondly, we have concerns about the necessary human resources stated by the authors. They indicate that 1.19 therapists are necessary to conduct a conventional therapy session, while 1 therapist is used to conduct a robotic session, regardless of the type of the robotic device. This stands in contrast with previously published data by Morrison [2] and Esquenazi et al. [3]. Morrison [2] indicated that 1 trainer is required for robotic locomotor training, while up to four trainers are needed for a manual locomotor training session. This difference is likely due to the fact that Morrison compared intensive, repetitive locomotor training assisted by a robot (robotic locomotor training, rLT) to similarly intensive, repetitive locomotor training assisted manually by physical therapists and physical therapy aids (manual locomotor training, mLT). Esquenazi et al. [3] compared mLT to rLT with the Lokomat on one hand and the G-EO on the other hand. They comment that one physical therapist was needed to conduct a Lokomat training, while manually assisted training and training with the G-EO required the help of more than one physical therapist, particularly in patients with profound weakness.

The articles considered by Carpino et al. all compare rLT in combination with conventional physical therapy to conventional physical therapy alone, but what exactly was done during the conventional therapy is not uniform. Most of the time, it did not consist of intensive mLT. In those cases where intensive mLT on a treadmill with physical therapist assistance is provided as a comparison treatment, the cost of the treadmill and support system must also be included for a complete picture. In Morrison et al.'s study, these costs were estimated at $100,000. Carpino et al. did not account for any costs of alternatives in the conventional therapy groups.

We would like to go even further than that and suggest that rLT should be conducted in a group setting. One of the big advantages of the advanced technologies is that training can be conducted with less direct supervision, allowing parallel sessions and hence increasing the amount of therapy sessions an individual therapist can provide. This in turn allows clinics to provide more longer and more intensive training sessions without increased costs as reported by Spiess and Colombo [4]. This is already done by many world leading rehabilitation clinics. We believe that when using advanced technology, it is important to leave traditional one-on-one therapy behind and exploit those devices' full potential by using them to increase therapy time and intensity for multiple patients. This will allow to improve outcomes without increasing the costs and hence increase financial efficiency.

## 3. Safety

Our last major concern about the article relates to the authors' statement about the limitations of exoskeletons devices. They propose that when the joints of the device are not properly aligned with the anatomical joints of the person using the device, undesired high forces pose a threat to the patient, as well as an obstacle to their movement. We agree that it is of utmost importance to correctly align the exoskeleton to the patient. However, we disagree strongly that setting up a patient improperly poses a threat to their safety. The exoskeleton Lokomat, due to its design, has integrated force sensors for the knee and hip joints. Interactions at the patient-machine interface are continuously measured, and the machine has safety features that stop the device immediately if forces are deemed unsafe for the patient. These mechanisms have undergone extensive testing and are a highly regulated process in the development of medical devices. In addition, as user training is central, users of the device undergo intensive training before being allowed to apply the device to their patients.

On the other hand, especially with patients with severe impairments, the lack of guidance at the hip and knee in end-effector type devices leads to a more movement variability and less precise walking pattern [5]. This in fact can pose a threat to patient care that can only be overcome by manual assistance or by using an additional knee brace. In summary, we strongly disagree with the statement by Carpino et al. [1] that exoskeletons pose a higher risk to the patient than end-effector device users.

## 4. Further Comments

The authors have reproduced the meta-analysis from the Cochrane review by Mehrholz et al. from 2013 [6] and have added 5 additional, newer articles. We would like to point out that this Cochrane review has also been updated and that the version from 2017 [7] by Mehrholz et al. includes two of the five articles that Carpino et al. have added by Forrester et al. [8] and Kim et al. [9]. Interestingly, Mehrholz et al. did not include the other three articles that Carpino et al. deemed “perfectly fit in terms of inclusion criteria for the patient and the type of trials with the papers included in the Cochrane review.”

Carpino et al. also indicate that they included “all articles in the more recent Cochrane review [7] and more recent studies by Kelley et al. [10], Calabroet al. [11], Forrester et al. [8], Kim et al. [9], and van Nunenet al. [12].” As Mehrholz et al. [6] included 23 trials in their 2013 version of the Cochrane review and Carpino et al. [1] indicate that they added 5, this should amount in a total of 28 studies; however, in the end, they state that they only included 26 trials, and it is unclear which two they excluded and why.

Mehrholz et al. [7] conclude that electromechanically assisted gait therapy in combination with physiotherapy increases the odds of walking independently, compared to physiotherapy alone. Carpino et al. [1], however, state that “robot-mediated therapy is more effective than the conventional one.” This is not the same statement, and it is important to distinguish between the two.

In summary, we are concerned about several aspects of the study by Carpino et al. [1]. We invite the authors to consider our concerns and potentially provide some updated data. We also invite all readers to carefully question the conclusion drawn in this study before integrating them into their decision-making process.
